# Supplementary figures and images for: High Tartronic Acid Content Germplasms Screening of Cucumber and Its Response to Exogenous Agents
Source: Foods. 2024 May 10;13(10):1484. doi: 10.3390/foods13101484 (PMC11120510; doi:10.3390/foods13101484)

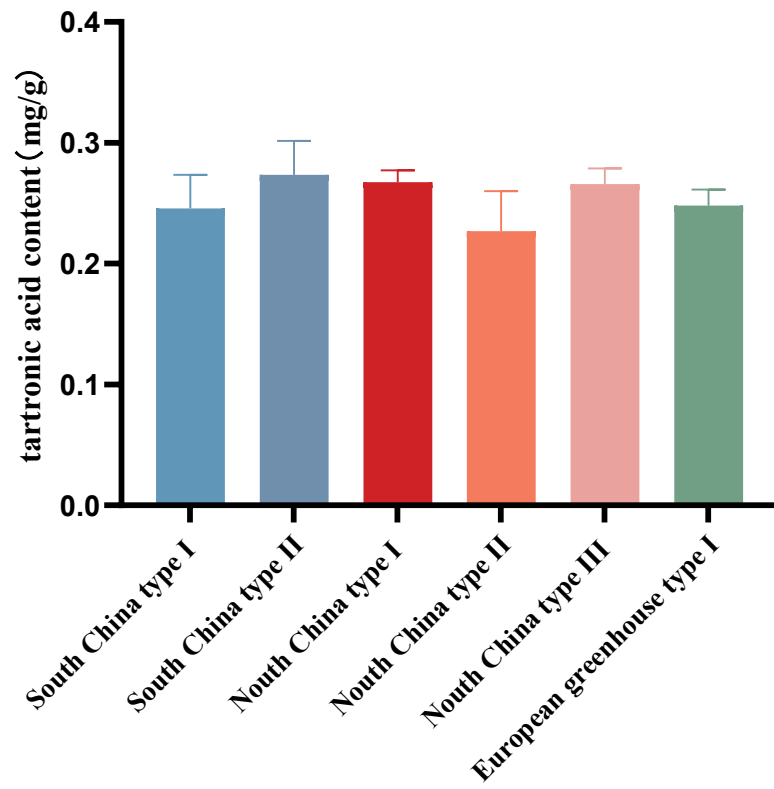

Figure S1. Tartronic acid content of existing commercially available cucumber varieties.

Supplement: Supplementary file 1 [file foods-13-01484-s001.zip › foods-2991819-supplementary.pdf]
